# Editorial: “Cancer Immunotherapy: Lights and Shadows”

**DOI:** 10.3389/fimmu.2015.00350

**Published:** 2015-07-07

**Authors:** María Marcela Barrio, Estrella Mariel Levy, José Mordoh

**Affiliations:** ^1^Fundación Cáncer FUCA, Centro de Investigaciones Oncológicas, Buenos Aires, Argentina; ^2^Fundación Instituto Leloir, Buenos Aires, Argentina; ^3^Instituto Alexander Fleming, Buenos Aires, Argentina

**Keywords:** cancer immunotherapy, immune checkpoint blockade, melanoma, mouse models, vaccines

Cancer immunotherapy has recently emerged as the fourth treatment modality, in addition to surgery, chemotherapy, and radiotherapy. These advances are the result of important discoveries in the field of regulation of the immune response, especially on the mechanisms which turn “on” and “off” immune responses (1, 2). A disease which has proved to be a canonical model to test therapeutic immunotherapy is the immunogenic cutaneous melanoma (3). So far, “passive” immunotherapy with monoclonal antibodies has outpaced “active” immunotherapy with antitumor vaccines (4, 5), and monoclonal antibodies which antagonize the “off” responses have been recently introduced in clinical practice (6, 7).

In this Research Topic containing nine articles, Aris and Barrio (8) present an updated review of current immunotherapeutic strategies and their combinations with oncogene-targeted therapy for cutaneous melanoma. Preclinical evidence, as well as emerging clinical results outlined in this review, might represent potentially powerful tools for cancer treatment. Besides, several monoclonal antibodies have been introduced into the clinic for cancer treatment. Among them, recently arrived anti-chemokine receptor antibodies are presented in a review by Vela et al. (9). The authors discuss the main achievements obtained with them to inhibit the interactions between cancer cells and their ligands. These antibodies hinder the interactions between chemokine receptors and chemokine signals delivered by different organs, thus preventing tumor cell survival, proliferation, adhesion, or migration that could result in metastatic spreading.

In spite of these recent successes, many unresolved practical and theoretical clues remain to be answered. The review by Madorsky Rowdo et al. (10) addresses relevant questions about the identity of the lymphocytes that eliminate tumor cells, their entry into tumor microenvironment, and parameters that could be used to determine the anti-tumor immune response. Also, the use of cancer vaccines to increase the lymphocytic tumor infiltration and the multiplicity of antigens that must be targeted to achieve significant clinical responses are discussed. Related to this issue, a Clinical Case Study reported by Aris et al. (11) shows histological evidence of the recruitment of immune cells induced by an anti-melanoma vaccine at the inoculation site, probably reflecting the early steps of the afferent immune response. Moreover, antitumor vaccination has been extensively developed in the last 15 years, even to target immune responses to hematologic tumors. Here, Di Stasi et al. (12) present the results obtained in the clinic with a peptide vaccination strategy that targets WT1 antigen in acute myeloid leukemia and myelodysplastic syndromes. Evidence of WT1-specific T cells induction that correlates with progression-free survival has been shown in several studies, encouraging further investigation to strengthen this cancer vaccination approach.

Regarding the development of adaptive antitumor immune responses, a basic assumption was that infiltrating anti-tumor immune cells were educated to recognize tumor antigens in secondary lymphoid organs, and were then recruited into the tumor microenvironment to exert anti-tumor activity. Here, Germain et al. (13) present evidence, focusing on the B cell compartment, suggesting that the immune response can also take place in tertiary lymphoid structures located in the areas of chronic inflammation as well as in cancer.

Dendritic cells (DC) play a pivotal role on the orchestration of the immune responses and are thus key targets in cancer vaccine design. In this Research Topic, three reviews address the role of DC in immunotherapy. First, recent findings in murine models regarding the anti-tumoral mechanisms of DC-based vaccination are reviewed by Mac Keon et al. (14). They focus on diverse issues, such as the antigen sources, the use of adjuvants, DC maturing agents, and the role of DC subsets in the antitumor response. In the human setting, Pizzurro and Barrio (15) present the main hot spots of the anti-tumor immune response, which are exploited with different DC-based vaccine designs. They focus on the processes taking place at the injection site, new adjuvants combinations, as well as lymph nodes homing to activate naïve lymphocytes and generating effector cells, and immune memory to control tumor growth. Finally, Pampena and Levy (16) discuss the participation of natural killer (NK) cells as alternative immune components that could cooperate in successful vaccination treatment. This article addresses the NK cell antitumor action sites and their role in DC-based cancer vaccines as determined by immune-monitoring in preclinical and clinical settings.

With no doubt, cancer immunotherapy is now a treatment modality that can change the reality for many cancer patients, achieving prolongation of their metastasis-free and overall survival. Scientific research over the last 20 years supports a key role of the immune system in the elimination of the disease, opening new roads for combinatorial treatments to be tested in the clinic. The contributions included in this Research Topic have exposed current lines of investigation, where clinical successes are recognized, but also a considerable lack of understanding of the mechanisms underlying such outcomes is also perceived. More research in the field is warranted to fully exploit the immune system as a therapeutic powerful tool for cancer patients.

## Conflict of Interest Statement

The authors declare that the research was conducted in the absence of any commercial or financial relationships that could be construed as a potential conflict of interest.
